# Geopolymer Materials for Bone Tissue Applications: Recent Advances and Future Perspectives

**DOI:** 10.3390/polym15051087

**Published:** 2023-02-22

**Authors:** Laura Ricciotti, Antonio Apicella, Valeria Perrotta, Raffaella Aversa

**Affiliations:** 1 Department of Architecture and Industrial Design, University of Campania, Luigi Vanvitelli, 81031 Aversa, Italy; 2 Advanced Material Lab, Department of Architecture and Industrial Design, University of Campania, Luigi Vanvitelli, 81031 Aversa, Italy

**Keywords:** geopolymer, bone tissue, biocompatibility, hydroxyapatite, biomaterials

## Abstract

With progress in the bone tissue engineering (BTE) field, there is an important need to develop innovative biomaterials to improve the bone healing process using reproducible, affordable, and low-environmental-impact alternative synthetic strategies. This review thoroughly examines geopolymers’ state-of-the-art and current applications and their future perspectives for bone tissue applications. This paper aims to analyse the potential of geopolymer materials in biomedical applications by reviewing the recent literature. Moreover, the characteristics of materials traditionally used as bioscaffolds are also compared, critically analysing the strengths and weaknesses of their use. The concerns that prevented the widespread use of alkali-activated materials as biomaterials (such as their toxicity and limited osteoconductivity) and the potentialities of geopolymers as ceramic biomaterials have also been considered. In particular, the possibility of targeting their mechanical properties and morphologies through their chemical compositions to meet specific and relevant requirements, such as biocompatibility and controlled porosity, is described. A statistical analysis of the published scientific literature is presented. Data on “geopolymers for biomedical applications” were extracted from the Scopus database. This paper focuses on possible strategies necessary to overcome the barriers that have limited their application in biomedicine. Specifically, innovative hybrid geopolymer-based formulations (alkali-activated mixtures for additive manufacturing) and their composites that optimise the porous morphology of bioscaffolds while minimising their toxicity for BTE are discussed.

## 1. Introduction

Although bone tissue possesses a high capacity for self-regeneration after injury, its physiology may change because of pathological conditions. In this regard, it is essential to employ innovative technologies to improve the healing processes of bone tissue [1,2].

As a connective tissue organ with mineralisation, bone plays a role in several crucial bodily processes, including tissue support and protection, motility, calcium and phosphate storage, and bone marrow housing. Their mechanical characteristics may change depending on the function and location [1]. Bone can be described as a nanocomposite made of water, organic substances (mostly collagen), and inorganic nanocrystalline hydroxyapatite (HA). An extracellular matrix (ECM) made of nanostructured proteins regulates the adhesion, proliferation, and differentiation of several cell types, including osteoblasts, bone-lining cells, osteocytes, and osteoclasts [2,3,4,5].

The bone tissue, from its macroscale to microscale structure, is shown in Figure 1.

Due to their shape and capacity to bond to the bone matrix (an example of the mechanism of bone cell proliferation within a geopolymer scaffold is reported in Figure 2), amorphous silicate-based materials have drawn much attention in the scientific study of the regeneration of hard tissues. Geopolymers have recently been studied as prosthetic biomaterials, and there are few academic papers on their use in BTE [7,8,9,10].

Geopolymers (alkali-activated materials) are amorphous systems with aluminosilicate bases produced by alkalinising natural or waste substances, such as metallurgical, industrial, urban, and agricultural wastes [7]. At a temperature below 100 °C, the aluminosilicate precursor powder combines with an activating alkaline solution of sodium and/or potassium hydroxides and silicates to create a ceramic amorphous matrix [7].

These materials are currently being investigated for their potential application in a wide range of scientific and industrial fields [8,9,10,11], including civil engineering (as cement and concrete), automotive and aerospace sectors, non-ferrous foundries, metallurgy–ceramics, building retrofitting, waste management, and art and decoration. They can reduce production-related energy use, greenhouse gas emissions, and environmental effects when used as cement and concrete components [7,8]. Traditional aluminosilicate concrete has several drawbacks, including limited chemical resistance to acids and salts, poor thermal and fire resistance, and considerable global carbon dioxide emissions [12], which can be overcome using geopolymeric formulations [13,14,15].

Geopolymer materials can be prepared as organic–inorganic composite materials and/or nanocomposite systems [16,17,18,19,20,21].

Although geopolymers exhibit important chemical–physical, mechanical, and morphological characteristics for BTE, their application in biomedical engineering is still limited. This limitation is mainly due to their low biocompatibility and osteoconductivity; however, the interest in using these systems in biomaterials has grown in recent years, as shown by the statistical analysis reported in Figure 3.

The results obtained from the Scopus database show increasing academic interest in geopolymer materials for biomedical applications. Many aspects should still be investigated and strengthened in this regard: (i) the careful selection and characterisation of aluminosilicate precursors because of the potential presence of heavy metals that could cause severe damage to health; (ii) the investigation of potential strategies to decrease the high alkalinity possessed by geopolymers, which severely limits biocompatibility; (iii) the study of the stoichiometry of the geopolymerisation reaction to finding the correlation between the Si/Al ratio and biocompatibility with the cellular microenvironment.

This paper evaluates the potential of geopolymer materials in biomedical applications by analysing published research articles’ results and comparing them with the features of the materials usually employed as bioscaffolds by focusing on the benefits and drawbacks. This review discusses the most recent developments in geopolymer-based materials that might be used in the biomedical industry. Critical concerns and obstacles are underlined. Applications and technological developments are discussed, along with potential progress and prospects.

## 2. Biological and Structural Material Requirements

This section reviews the main characteristics materials should possess for bioscaffold use in the biomedical field.

### 2.1. Biological Features

Collagen, water, and several minerals combine to produce the bone matrix, which is divided into organic and inorganic phases. The organic and inorganic components comprise the matrix, comprising around 70% hydroxyapatite (HA) in the inorganic phase and 30% collagen in the organic phase by weight of the whole bone [22]. Scaffolds should prevent potentially harmful reactions and have biological properties that are comparable to those of native bone.

Biocompatibility is the most crucial requirement for a biomaterial to be considered for usage as a possible tissue replacement. When used as bone scaffolds, materials should promote osteogenesis and minimise or prevent adverse consequences, such as the deterioration of native and healthy tissues [23,24] and the body’s inflammatory response, because such materials can be seen as foreign and be considered a threat.

For these reasons, bone scaffolds must be subject to strict sterility constraints and maintain their structural integrity during sterilisation phases [25]. Moreover, ideal BTE scaffolds should allow cells to adhere to biomaterial surfaces, promoting their subsequent growth (see Figure 4).

Furthermore, the osteoconductive property (namely, the capacity of bone-forming cells to migrate over a scaffold and gradually substitute it with new bone over time) is another crucial aspect to consider when the quality and effectiveness of a biomaterial is being determined [24,26].

In addition, bioscaffolds could be bioresorbable and monitorable in their degradation process to support new tissue formation; this feature is closely related to biocompatibility. Biocompatibility also involves designing and using materials while respecting the equilibrium between their strength and integrity with the necessary bone tissue ingrowth over time. Thus, bioresorbable scaffolds should exhibit mechanical properties that are as comparable as possible to the natural bone but, at the same time, promote degradation phenomena over time (with an easily monitorable degradation rate) for completing tissue growth and maintaining structural support [27,28,29,30,31].

Biodegradability allows bioabsorption so that the body can naturally break down the system and absorb it without creating pH changes in the cellular environment. This may result in the leaching of hazardous byproducts during the bioscaffold in vivo implantation and biodegradation processes, affecting the pH values at or close to the implant site [31,32,33]. Alterations in pH levels may slow cell division, reduce cell reproduction, and ultimately prevent growth [31,33]. Designing bioscaffolds in line with the structure and makeup of healthy and native bone tissue is crucial to avoid this problem.

Finding all of the described characteristics contemporaneously in a single material (which should emulate the composition, structure, and physiology of natural bone tissue) is very difficult. Thus, it is fundamental to develop biomimetic materials, composites, and coatings to replicate bone tissue features as much as possible [28,29,30,31,32,33,34,35,36,37,38,39,40].

### 2.2. Structural Features

Bones have different fundamental bodily roles: structural support and protection, locomotion, and reservoir action for several essential minerals [22].

This characteristic tissue exhibits a unique hierarchical structure compared to other typical tissues in the human body [26]. The bone structure can be divided into three dimensions: macro (compact bone and cancellous, cortical, or trabecular bone), micro (Haversian canals, concentric lamellae, and osteons), and nano (HA crystals, collagen fibrils, and other minerals).

Bone is an anisotropic material with a unique structure not found in any other tissue. This system is complex and challenging to recreate because it exhibits different measured values in the longitudinal and transverse directions of compressive strength and the elastic modulus [26].

An implanted scaffold for bone tissue must support human function under normal compressive pressure. The need to use biomimetic scaffolds with mechanical characteristics that enable them to fulfil structural roles while remaining flexible enough to prevent shear fracture under continuous compressive stresses becomes crucial [32,38,40].

Strength, which is one of the most critical characteristics for load resistance over a long time, and structural bone grafting, representing the capability of cells to spread across, adhere to, and bioaccumulate novel minerals as a replacement for bone structures, are fundamental key factors for BTE structures [30,31,32,38].

In addition to mechanical strength characteristics, bioscaffolds must possess high porosity (porosity is the fraction of voids in a solid) to perform their functions to the best of their ability. Thus, the right balance between porosity (worsening mechanical strength) and mechanical properties supporting the bone’s structural functions is needed [30,31].

Another critical factor is surface morphology (closely related to porosity), which can affect the mechanical resistance and the development of new bone tissue. Porosity is a crucial element in the growth of novel tissue thanks to its role in promoting cell migration and angiogenesis, driving the transfer of essential nutrients [30].

To improve the surface morphology, it is possible to carry out coating processes that enhance cell proliferation and promote the incorporation of the biomaterial within the healing location [31].

Finally, parallel to the bone scaffold’s mechanical strength and load-bearing capacity, there is a pressing need to promote the osteoconductive ability of the bone scaffold [32,38].

## 3. Materials

This section describes the main classes of materials usually used as bioscaffolds in the biomedical field, along with an analysis of their positive characteristics and limitations.

Due to the complicated nature of bone, there is a need for manufactured replacements that consider every aspect of the bone structures. Scientific research allowed us to investigate different materials, such as metals, ceramics, polymers, and sometimes hybrid combinations of other materials.

The ideal biomimetic material for a bone scaffold should be biologically compatible, osteoconductive, biodegradable, sufficiently porous, workable, and mouldable, allowing an idoneous environment for the osteogenesis process, with suitable mechanical properties to bear critical loads and mimic structural bone tissues [23,26,31,32,33].

Usually, biodegradable materials are also biocompatible but are not strong enough to create implantable scaffolds.

Polymers, ceramics, and metals are the most used biomaterials in bone regeneration research [38]. The statistical distribution of the leading studied biomaterials in publications retrieved from a search in the PubMed database is reported in Figure 5a.

Collagen, and other natural materials, cannot be used alone but as additives or coatings to improve the biocompatibility of the bioscaffold and improve the osteogenic response of cells [35,36].

A coating can be also applied to lessen the material’s toxicity. HA and collagen are two of the most often utilised biocoatings. Some scaffolds can be applied to achieve biocompatibility and support transporting growth factors and cells [31,32,38]. For instance, by adding physiologically appropriate coatings to metal-based bioscaffolds, it is feasible to enhance compatibility and osteogenic differentiation while maintaining the physical qualities of the scaffolds [31,38].

### 3.1. Polymers

Polymeric materials have several interesting properties, making them versatile and easy to use as bioscaffolds in bone prostheses [32,40,41].

Usually, polymeric materials can be easily synthesised, and their structures can be modified and adapted to different applications. In addition, they are workable, processable, and printable (with 3D printing) and can be customised into desired geometries [37,40]. In addition, the porosity (porosity percentage and size of the voids) can be easily modulated [41,42,43,44].

Polymers used for BTE can be of natural or synthetic origin. They can be divided into three main classes: polynucleotides (such as DNA and RNA), proteins (such as collagen, silk, and fibrin), and polysaccharides (such as chitosan, cellulose, and glycosaminoglycans or GAGs) [41,42].

Depending on their use (for long-term implantation or dissolvable grafts), polymers can be further categorised as either nondegradable or degradable [42]. Polyglycolic acid (PGA), polylactic acid (PLA), and their copolymer, poly(lactic-co-glycolic acid) (PLGA), are examples of synthetic degradable polymers [31,43].

Natural polymers include several crucial characteristics that make them adaptable components for bioscaffolds: high cellular attachment rates, biological recognition, and strong support for ECM deposition [44]. However, they have some issues with immunogenicity or triggered immune reactions due to(potential contaminants in the polymer chains [45,46].

Synthetic polymers may be easily moulded into complicated shapes due to their diverse chemical and physical characteristics. Unfortunately, because PLA and polycaprolactone (PCL), the two most prevalent polymers used in bone scaffolds, are hydrophobic, surface treatments, coatings, or the inclusion of other materials is required to increase cell adhesion [46].

Finally, although polymers are frequently readily available, they often require enhancements for bioscaffold use, such as using additional materials to optimise some of the scaffolding criteria they do not meet owing to poor mechanical qualities.

### 3.2. Metals

When used in bone scaffolds, metals and their alloys, such as stainless steel, titanium (Ti), and cobalt (Co), demonstrate the most important physical and mechanical qualities.

Due to their unusual qualities, such as biocompatible properties, exceptional corrosion resistance, and mechanical capabilities, these materials have been utilised as bioscaffolds for many years (this type of material has been used for 100 years [47]).

Conversely, they may need to be structurally stronger to sustain complex geometries when processed to obtain the appropriate porosity for bone bioscaffold applications. Moreover, they can rarely be used alone as bone scaffolds [40,48]. Generally, metallic materials are applied as support plates when bone injuries need stabilising to achieve healing. This technique, dating back a hundred years, is known as plate osteosynthesis or internal plate fixation. Although this technique is fundamentally supportive for the complete healing of the bone lesion, it shows some limitations: the use of uncomfortable and painful supports such as screws, nails, and wires and the possibility of having fracture misalignment during the convalescent period due to the rigidity of the metal support [48,49].

Metallic materials have the most similar mechanical properties to bone structures, especially when compared with other types of materials. However, they have excessive stiffness when compared with bone fibres, and thus, a failure to absorb the energies involved during physical and mechanical stresses within the human body is observed. This generates a failure of the metal prosthesis [32,50].

Many metals release toxic substances and byproducts during degradation reactions and can cause harm to the native bone tissue [31,51]. In addition, the degradation reactions of several metals are easier to regulate or estimate with a suitable surface coating [31]. For these reasons, metallic materials are not ideal for making bone bioscaffolds [51].

### 3.3. Ceramics

Compared to other materials used in bone engineering, ceramic materials appear to have the best biocompatibility. Generally, bioceramics can provide the maximum degree of cell development and adhesion for bone cells (osteoprogenitor cells) and match the composition and structure of bone tissue [52,53].

Because of these unique properties, bioceramics may bond with the nearby living and developing tissue immediately around insertion sites, significantly enhancing their structural function and serving as a bone scaffold [54]. Essentially, when these materials are tested in vivo, they are highly similar to healthy bone tissue.

**Figure 6:**
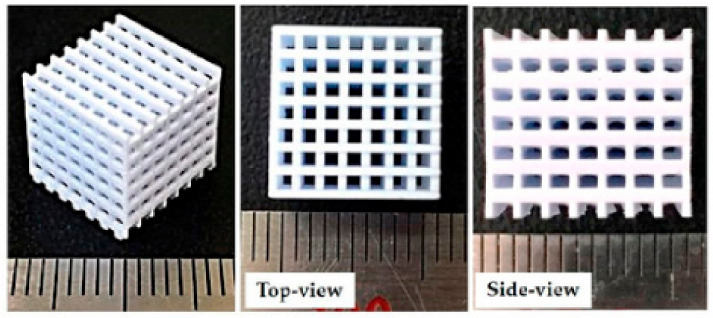
CaP scaffold with porous structure [60].

**Figure 1:**
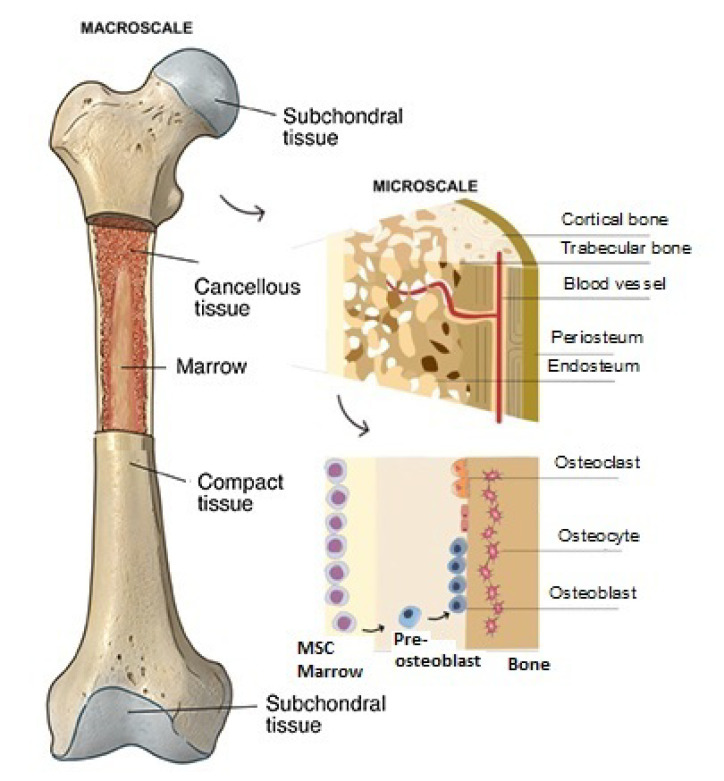
The macroscale and microscale (mesenchymal stem cells (MSCs)) of femur bone tissue [6].

**Figure 2:**
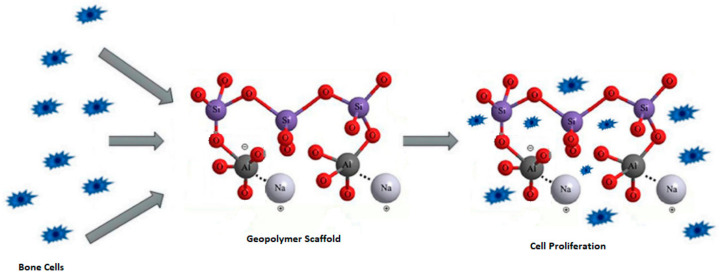
Outline of bone cell proliferation within geopolymer scaffold.

**Figure 3:**
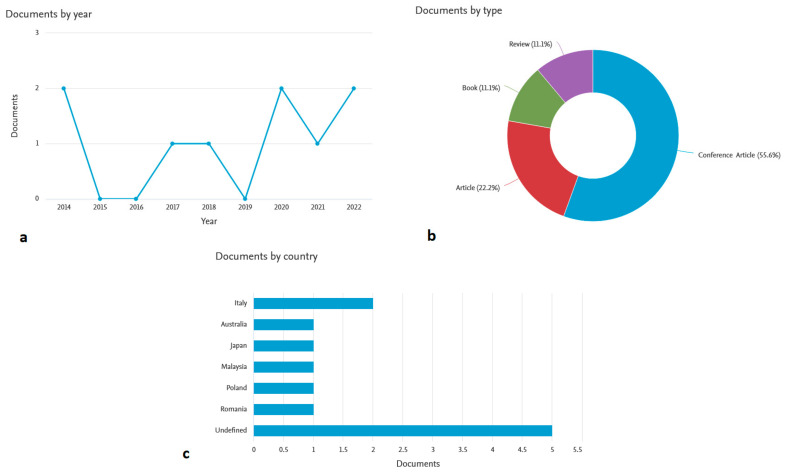
Statistical analysis of published articles on the topic “geopolymers for biomedical applications”: (**a**) number of publications per year, (**b**) types of publications, and (**c**) countries actively involved in article publication. The data were extracted from the Scopus database on 23 January 2023.

**Figure 4:**
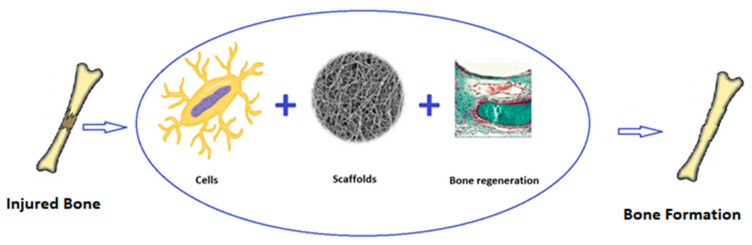
A flow chart illustrating the bone regeneration process [25,26].

**Figure 5:**
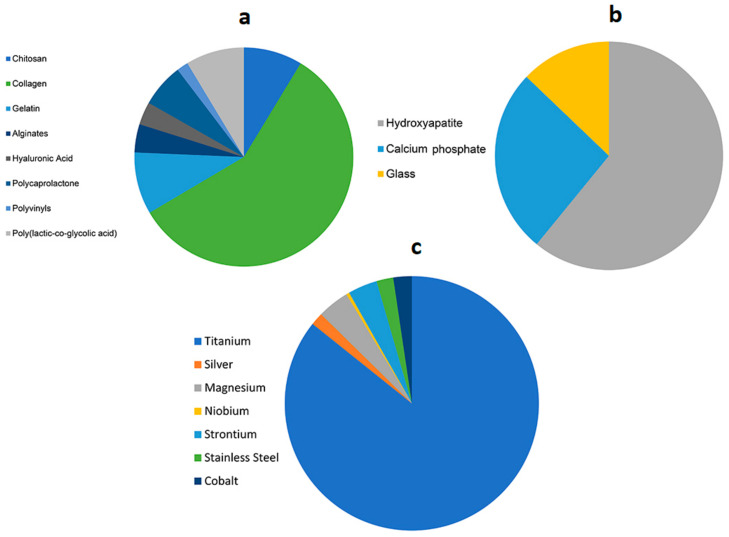
Graphs showing biomaterial distribution in bone regeneration publications extracted from a search in the PubMed database in 2020 polymers (**a**), ceramics (**b**), and metals (**c**).

Bioceramics are frequently divided into three categories: bioactive (e.g., HA and Bioglass^®^), bioinert (e.g., alumina and zirconia), and biodegradable (e.g., tricalcium phosphate and calcium sulphate). Calcium phosphates (CaPs—see Figure 6), including HA, calcium sulphate, calcium carbonate, and tricalcium phosphate (TCP), as well as well-known bioactive glasses (BAGs) such as 45S5 Bioglass^®^, are some of the bioactive ceramics that are most frequently employed in bone engineering [55,56].

Although bioceramic materials have high biocompatibility, these systems do not have torsion and tensile strength compatible with natural bone structures, limiting their use [57]. In addition, many ceramic bioscaffolds possess lower biosorption capacity than many natural polymers used in bone engineering, creating potential damage during bone growth [58]. Some studies have been conducted to speed up the degradation processes of slow-degrading ceramics, notably HA, by adding structure-destabilising agents (via the addition of ions) to the material’s structure [59].

### 3.4. Composites and Hybrid Materials

Research has focused on composite materials because of the need for engineered bone scaffolds to have a variety of biological and physical characteristics (such as biological and mechanical biocompatibility, biodegradability, bioactivity, porous architecture, and appropriate structural properties) that cannot be achieved with the use of a single type of material [61,62,63,64,65].

Hybrid ceramic–polymeric nanocomposites based on hydrophilic polymers and amorphous nanosilica particles are good candidates for biomimetic materials in BTE [62,63]. The last few years have witnessed a significant increase in the understanding of organic and inorganic hybrid nanocomposite mechanisms for improving their physicochemical properties, enabling them to be used as advanced biomaterials translated into clinical applications [64,65,66]. The positive effect of the ceramic nanosilica content on the hybrid material nanosilica biocompatibility and cytotoxicity was described previously [62,66].

In particular, new bone scaffold materials with improved biocompatibility, non-immunogenicity, and non-toxicity, with controlled degradable absorption and degradation rates that best match the formation of new bone [31,62,67], have been described as presenting adequate porosities and structural characteristics to form three-dimensional networks with a large surface-to-volume ratio able to host osteogenic cells and growth factors. Enriching the porous hybrid scaffold with bioactive molecules, such as extracellular matrix proteins, adhesive peptides, growth factors, and hormones, positively enhances its physiological, mechanical, and biological properties [62,63,68,69,70].

Bone remodelling healing processes can be strongly favoured by using biocompatible and biomechanically active hybrid nanocomposites “designed” to reproduce bone-compatible and biomimetic structural characteristics.

**Figure 7:**
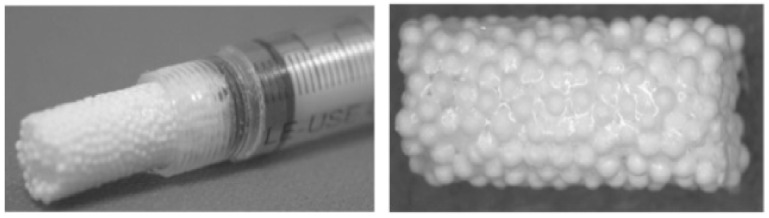
A 3D-formed composite of β-tricalcium phosphate beads and alginate [61].

An example of a scaffold made of composite material is shown in Figure 7. To generate new, specialised materials with enhanced physical, mechanical, and biological qualities compared with the starting materials, composite materials mix two or more materials having disparate physical and biological properties.

Numerous capabilities, such as enhanced cell adhesion, bioactivity, mechanical characteristics, and processability, have improved. Metals, ceramics, and polymers can all be used to create composites for bone tissue [62,63,64,65,66,67,68,69,70]. Osteoconductive materials based on functional composites have also been developed [62,69].

In BTE applications, scientific research has demonstrated improvements in cell attachment and mechanical strength in polymer-based bioscaffolds, osseointegration and bioactivity in metal-based bioscaffolds, and toughness in bioceramic-based bioscaffolds by combining different biomaterials [38,62,63,68,69,70,71,72].

Thanks to composite materials, engineered bone tissue prostheses have significantly improved many physical, mechanical, and biological characteristics (such as mechanical strength, bioactivity, osteointegration, and cellular attachment) [38,62,62,63,64,65,66,67,68,69,70,71,72]. However, because the unique features of bone structures have yet to be completely replicated in the laboratory, study and inquiry are still needed to understand the high complexity of genuine bone.

The leading biomaterials, together with their properties and fields of application, are shown in Table 1.

## 4. Geopolymer Materials

### 4.1. Synthesis and Applications

A class of aluminosilicate materials referred to as geopolymers are produced by an inorganic polycondensation reaction (also known as “geopolymerization”) between solid aluminosilicate precursors and alkaline solutions such as sodium hydroxide (NaOH), potassium hydroxide (KOH), sodium silicate (Na_2_SiO_3_), or potassium silicate (K_2_SiO_3_) or highly concentrated aqueous alkali hydroxide.

Usually, active silicon and aluminium are abundant in the raw materials used to make geopolymers. Generally, they consist of metakaolin and/or the byproducts of various industrial processes, such as fly ash, bottom ash, red mud, biomass ash, steel slag, volcanic ash, waste glass, coal gangue, diatomite, bauxite, high-magnesium nickel slag, etc.

The most significant addition to the understanding and scientific study of geopolymer materials was made by Davidovits [73]. By reacting natural minerals containing silicon (Si) and aluminium (Al), such as slag, clay, fly ash, pozzolan, and an alkaline activator under mild conditions (below 160 °C), he created the first inorganic geopolymer material in the 1980s [74].

**Figure 8:**
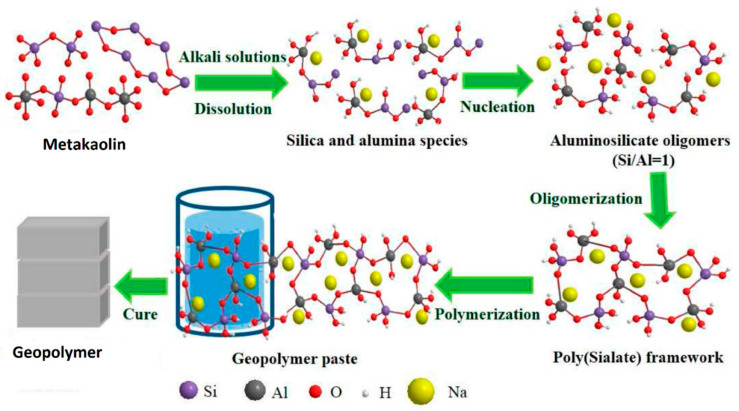
Reaction mechanism of a metakaolin-based geopolymer material [80].

The geopolymerisation reaction process may be broken down into three main steps (an example scheme is presented in Figure 8) [8,75,76,77,78,79,80,81]:

1. The dissolution of aluminosilicate materials in the concentrated alkali solution forms free silica and alumina tetrahedron units.
2. The condensation process between alumina and silica hydroxyl results in an inorganic geopolymer gel phase. This process causes water to leave the structure.
3. The developed three-dimensional silicoaluminate network hardens and condenses.

In aluminosilicate materials with a high degree of geopolymerisation, a high dissolution rate of Si^4+^ and Al^3+^ ions is found at high pH values (NaOH concentration > 10 M) [81,82].

The curing temperature is also crucial for the geopolymerisation process. The temperature accelerates the dissolution response of raw materials, and temperatures above room temperature (60–80 °C) are ideal for geopolymerisation [82].

Moreover, to create novel materials for cutting-edge technological applications, geopolymers can be functionalised, created as organic–inorganic hybrids, or combined with other materials to form composites [13,14,15,16,17,18,19,20,21].

Geopolymer paste can add the organic phase in a liquid or solid form, such as powder, fibres, or particles [83,84,85,86,87]. Due to the chemical incompatibility between strongly polar aqueous and apolar organic phases, adding a second liquid to a geopolymer that is non-miscible with water is particularly challenging. In particular, the organic component can be added using several methods and at various phases of the production of the composite: (i) Direct method. The solid precursors first dissolve in the alkaline aqueous solution to create the paste slurry. Before the system hardens, the organic phase is immediately absorbed into the slurry while being vigorously mechanically mixed. (ii) Method of pre-emulsification. First, the organic component emulsifies while the activating solution still lacks solid precursors. The solid precursor is added to the stable emulsion of the organic phase in the aqueous activating solution to start the paste-hardening process. (iii) Process of solid impregnation. Before being added to the geopolymer slurry, the organic phase is impregnated on a solid powder (either the aluminosilicate precursor or a specific adsorbing powder) and added to the alkaline activating solution.

The two primary geopolymer applications are those with traditional physical and mechanical qualities and those for functional and advanced applications.

Geopolymers in the first category can find applications in building, construction, repair, restoration, marine construction, pavement base materials, 3D printing, high-temperature and fire-resistant materials, and thermal and acoustic insulation. Special applications include heavy-metal pollution immobilisation, pH regulator materials, catalysts, conductive materials for moisture sensor applications, and thermal storage [88,89,90,91,92,93,94].

Functional applications can be employed for buildings in specific industries, such as fire prevention structures, insulation walls, and nuclear power plants. These include fire prevention, isolation, heat preservation, and adsorption of hazardous ions [95,96,97,98,99,100,101,102].

### 4.2. Applications in the Field of Biomaterials

Due to their capacity to adhere to the bone structure, geopolymer materials have attracted more interest in recent years in the field of hard tissue regeneration [102,103,104,105,106,107,108,109,110,111,112,113,114,115,116,117,118,119,120].

To minimise toxicity towards the tissues, using geopolymers in biomaterials calls for some fundamental characteristics, such as an aluminium silicate source free of heavy metals and/or other contaminants and biocompatibility that depends on the pH levels and the amount of aluminium released [110,120].

**Figure 9:**
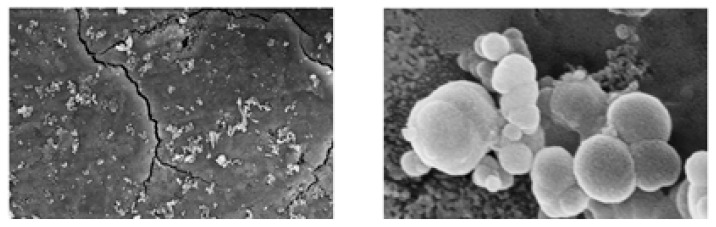
SEM micrographs of geopolymer samples soaked in simulated body fluid show the formation of some globular hydroxyapatite crystals [103].

A high-mechanical-strength geopolymer matrix was studied by Catauro et al. (compressive strength of 50 MPa) [103]. Geopolymer samples were immersed in a simulated bodily fluid (SBF) to assess their bioactivity, and the layering of hydroxyapatite (hydroxyapatite formation is considered a critical index for scaffold biocompatibility) on their surface was examined using SEM characterisations (see Figure 9). However, only modest bioactivity was found.

**Figure 10:**
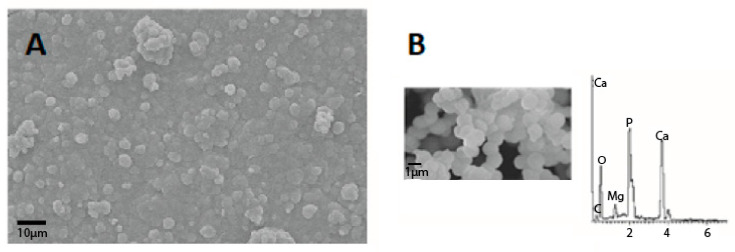
SEM micrographs of the geopolymer–Portland cement composite soaked in simulated body fluid for 28 days with globular hydroxyapatite crystals formed on the surface (**A**); EDS analysis of hydroxyapatite formed on geopolymer surfaces after soaking in SBF (**B**) [104].

Pangdaeng et al. [104] studied calcined kaolin–white Portland cement geopolymer as a possible biomaterial. In this instance, the mixture had a 28-day compressive strength of 59.0 MPa and contained 50% white Portland cement and 50% calcined kaolin. Still, as shown in Figure 10, only a small number of hydroxyapatite particles were formed on the surface of the geopolymer–Portland cement composite, indicating low bioactivity.

It is also important to note that high-temperature treatments have been shown to encourage the release of water molecules (the removal of absorbed water molecules, up to 100 °C, or differentially coupled, free water molecules inside the pores). When structural water and water bonded in nanopores are removed at high temperatures from the silicate matrix, the porosity of the final geopolymer material increases [105].

**Figure 11:**
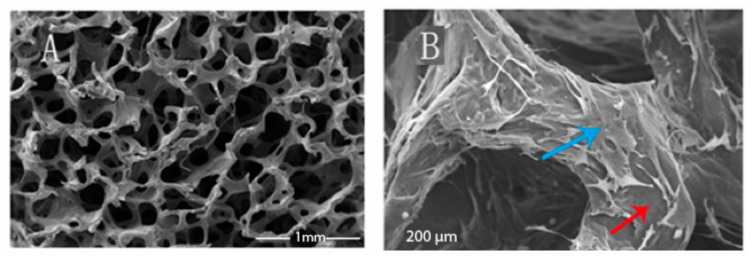
SEM micrographs of human porous bone tissue at low (**A**) and high magnifications (**B**) with osteoblast cell adhesion and spread (red and blue arrows [106].

The porosity of the geopolymer matrix is an essential key factor in increasing the biocompatibility of aluminosilicate systems by promoting bone tissue growth. The microstructure of the porous geopolymer appears very similar to bone tissue (see Figure 11).

The bone graft’s porous nature is crucial to biofactors’ distribution and tissue volume maintenance [107]. Because the microarchitecture of three-dimensional scaffolds regulates cell migration behaviour via junction interactions, the pores must have interconnected structures to allow for cell growth and migration. Finally, it is recognised that the size of the pores affects the vascularisation, infiltration, and cell attachment of the bone transplant [108].

As was already indicated, in addition to porosity, the pH value, which is well known to be relatively high in the aluminosilicate matrix, is a crucial factor in the biocompatibility of geopolymers. Thus, managing its high pH value is one of the critical factors needing careful attention for an alkaline geopolymer’s biological application.

Furthermore, a fundamental restriction on using geopolymers in biomaterials is the proportion of “free” aluminium, which may cause significant toxicity [109]. Lowering the quantity of aluminium used in the geopolymerisation reaction is an effective strategy to reduce toxicity.

**Figure 12:**
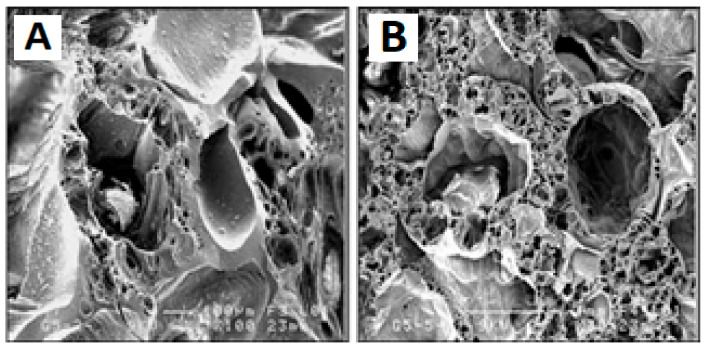
SEM micrographs showing the microstructure of geopolymer material subjected to heat treatment at 250 °C (**A**); at 500 °C (**B**) [110].

As possible biomaterials, Oudanesse et al. [110] investigated amorphous geopolymers of the potassium-poly(sialate)-nanopolymer type with a mole ratio of Si:Al = 3:1. After, a heat treatment at 500 °C was carried out to lower the geopolymer matrix’s alkalinity from pH 11.5 to pH 7.1, as demonstrated in Figure 12, excellent porosity for biological compatibility was attained.

The authors report that the high-temperature treatment made it possible to positively impact the mechanical strength and the geopolymer network, with the possible stabilisation of the free alkali present in the aluminosilicate matrix.

In this context, it is essential to note that porous geopolymers can be created at high temperatures or by utilising specific foaming agents, such as hydrogen peroxide, metallic Al or Si powders, or both [111,112]. Porous materials with pore sizes ranging from nanometres to a few millimetres and a total porosity of up to 90% [111,112] can be obtained without using high-temperature treatments (such as burnout of organics and sintering).

This is particularly true if foaming agents are included in the geopolymer paste before it is condensed. Due to its simplicity and low cost, the direct foaming approach in additive manufacturing has recently been tested to produce high-strength geopolymer foams [113,114,115,116,117,118].

Other foaming techniques have also been developed, including gel casting, saponification, and foaming agents that include different oils. These enable the creation of extended and cellular structures with pores ranging from the meso- to the macro-range for use in wastewater treatment, catalysis, and thermal and acoustic insulation, among other fields [15,95,102].

**Figure 13:**
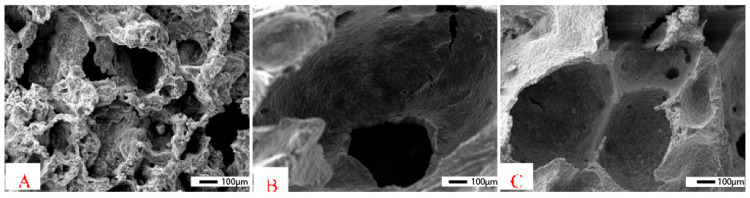
SEM micrographs of metakaolin-based porous geopolymers using aluminium powder and cured at 80 °C for 4 h, Aluminium powder/pre-geopolymer formulation ratios: 1:1 (**A**), 1:1,5 (**B**), and 1:3 (**C**) [119].

In this regard, Faza et al. [119] studied metakaolin geopolymer-based foams obtained as potential scaffolds for bone substitutes. The authors developed metakaolin-based porous geopolymers using aluminium powder as a foaming agent. Aluminium powder was added to a combination of metakaolin, sodium silicate, and sodium hydroxide in the following ratios: 1:1, 1:1,5, 1:2,5, and 1:3. The samples were hardened in an oven at 80 °C for four hours. SEM micrographs (see Figure 13) show a sample morphology like human spongy bone (with a size void of 80–400 µm).

By combining varying concentrations of hydrogen peroxide (H_2_O_2_) as a foaming agent from 0 to 6 vol% and heat treating it at 500 °C for 1 h, Sayed et al. [120] reported the synthesis of foamed geopolymer structures. The formulation with 4.5 vol% H_2_O_2_ was the best regarding the examined samples’ open porosity and mechanical characteristics.

Geopolymer samples showed an open porosity of 71 vol% and compressive strength of 3.56 MPa, which are suitable for 3D scaffolds used in biomaterial applications.

**Figure 14:**
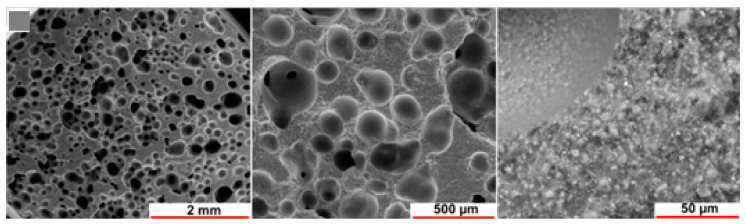
SEM micrographs at increasing magnifications of foamed geopolymer structures using H_2_O_2_ at 4.5 vol% and heat-treated at 500 °C for 1 h [120].

The microstructure of the selected composition is shown in Figure 14.

It is important to note that the foamed geopolymer’s internal microstructure and bone tissue’s morphology are highly comparable (see Figure 11). Finally, after 28 days of immersion in simulated bodily fluid solutions, the pH level of the geopolymers remained near the physiological value. The geopolymer foams showed bioactivity in an in vitro investigation, as shown by apatite particles appearing on their surface after 28 days of immersion in a solution simulating bodily fluids.

Using two different preparation techniques, Catauro et al. [121] studied the application of geopolymers with the composition H_24_AlK_7_Si_3_1O_79_9 and a ratio of Si/Al = 31. In the first case, the alkaline activating solution was made using KOH in the form of pellets in a potassium silicate solution. In the second case, a solution of KOH 8M was added to the potassium silicate solution. Varied water contents were employed, and only some manufactured samples were heated.

The study demonstrated that the mechanical characteristics of geopolymer materials are influenced mainly by the changing experimental conditions (chemical composition and curing temperature). It was reported that using KOH 8 M to generate the alkaline activating solution and heating it to 65 °C was the best method to improve mechanical properties (i.e., compressive strength up to 1.95 MPa). According to the same authors, heat-curing eliminates the water generated during the condensation stage and enhances the geopolymerisation reactions. However, increasing the aqueous phase could lead to uncontrolled void nucleation during the geopolymerisation stage. The geopolymer bioactivity was evaluated by monitoring the apatite-forming ability on the aluminosilicate matrix surface after being soaked in SBF for 21 days.

**Figure 15:**
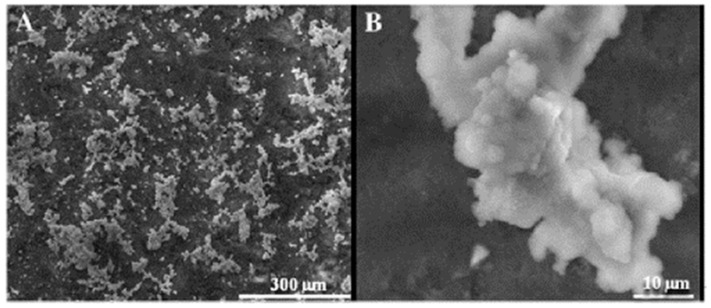
(**A**) SEM micrographs of the GEO-TA’ sample after soaking in SBF for 21 days and (**B**) detail at high resolution of a hydroxyl-apatite crystal formed on the geopolymer sample [121].

The microstructure of the GEO-TA’ sample is shown in Figure 15.

The authors concluded that the hydroxyl-apataversaite [Ca_10_(PO_4)6_(OH)_2_] formation was detected on the surfaces of all geopolymers after their immersion in an SBF solution for 21 days and thus, all samples with both alkaline activating solutions and treatments at room temperature and high temperature (hydroxyapatite crystal growth was found to be independent of the type of alkaline activating solution and heat treatment).

According to the literature [122,123], hydroxyl groups on the surfaces of silica glasses and ceramics encourage the formation and nucleation of hydroxyl-apatite. This process is enhanced when the materials include cations, such as Na^+^ or K^+^ ions. These systems can release cations via a cation-exchange mechanism with H_3_O^+^ ions in SBF to form Al-OH and Si-OH groups on their surface; a rise in pH is observed during this reaction in the SBF solution, and this results in the dissociation of Al-OH and Si-OH groups into the negatively charged units Al-O and Si-O. These anions boost the positive charge on the surface when they connect with the Ca^2+^ ions already present in the fluid. The negative charge of the phosphate ions and the simultaneous binding of Ca^2+^ cations create an amorphous phosphate, which results in the synthesis of hydroxyl-apatite [Ca_10_(PO_4)6_(OH)_2_].

An interesting study published by Pangdaeng et al. [124] focused on improving geopolymer bioactivity through CaCl_2_.

Specifically, calcined kaolin (metakaolin), a sodium hydroxide (NaOH) solution, a sodium silicate solution (as an alkaline activating solution), and heat curing (60 °C for 24 h) were used to create the geopolymer material. The geopolymer samples were treated using the soaked-treatment approach, which accelerated apatite precipitation and slowed the rise in pH. A CaCl_2_ solution was used as an ion-exchange agent. After preparation, the samples were treated in the CaCl_2_ solution for 24 h at 23 °C.

The contact of the calcium chloride solution with the geopolymer increased the calcium ion absorption. It improved the chemical interaction between aluminosilicate components via a mechanism like that observed for cementitious materials [125,126].

The surface of the geopolymer is subjected to an increase in its hardness due to a cation-exchange mechanism between Ca^2+^ and Na^+^ ions. This phenomenon causes calcium precipitation on the surface because of the improvement in surface hardness.

**Figure 16:**
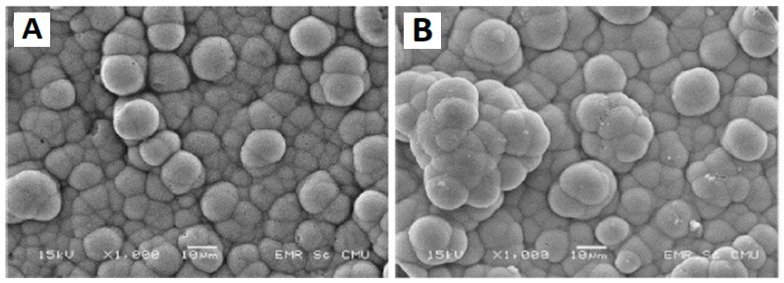
SEM micrographs of the geopolymer surface treated with CaCl_2_ solution after soaking in SBF for 3 days (**A**) and 28 days (**B**) [124].

The microstructure characterisation of the geopolymer surface after soaking in an SBF solution (at 3 and 28 days) is shown in Figure 16.

The geopolymer material surface’s microstructure showed a dense hydroxyapatite structure with a thickness of around 15 µm (Figure 16B).

In research on the effects of the curing time and temperature on geopolymer systems made from hydroxyapatite and calcined kaolin powders as raw materials, Sutthi et al. [127] presented their findings. In particular, the effects of curing hydroxyapatite and calcined kaolin at different temperatures (40 °C, 60 °C, and 80 °C) and for different times (2, 7, 14, 21, and 28 days) were examined.

A statistical analysis was conducted to determine the extent of each variable’s effect. The authors concluded that as the curing time and temperature increased, the compressive strength of the geopolymer materials also dramatically increased.

The best compressive strength measurement (37.8 MPa) was attained after 28 days of curing at 80 °C. The best experimental conditions that led to high compressive strength were also optimal for bioactivity and, thus, for applying geopolymer systems as bone substitute materials.

Tippayasam et al. [128] investigated the effect of calcium hydroxide addition to geopolymer material formulations. Moreover, their mechanical properties were investigated, and chemical–physical and bioactivity characterisations were carried out. The potential bioactivity of the aluminosilicate systems was studied after soaking them in SBF solution for 28 days.

**Figure 17:**
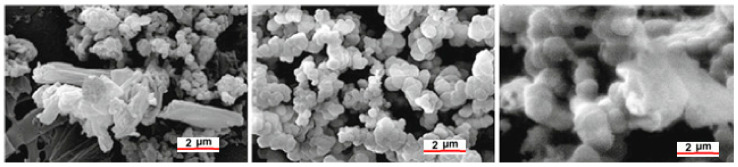
SEM micrographs of apatite layer formation at 20,000 magnification (reference bars 2 μm) on the top surface of geopolymers (Ca/k: 3.82) soaked in SBF for 14, 28, and 90 days [128].

The SEM micrographs in Figure 17 demonstrate that when the quantity of Ca(OH)_2_ increases, more bone-like hydroxyapatite layers tend to develop on the surface of geopolymers.

Radhi et al. [129] produced a foamed geopolymer material and evaluated its potential application as a bone substitute. A metakaolin-based system employing various ratios of olive oil and hydrogen peroxide as foaming agents was prepared, resulting in different porosity percentages and sizes of geopolymers.

**Figure 18:**
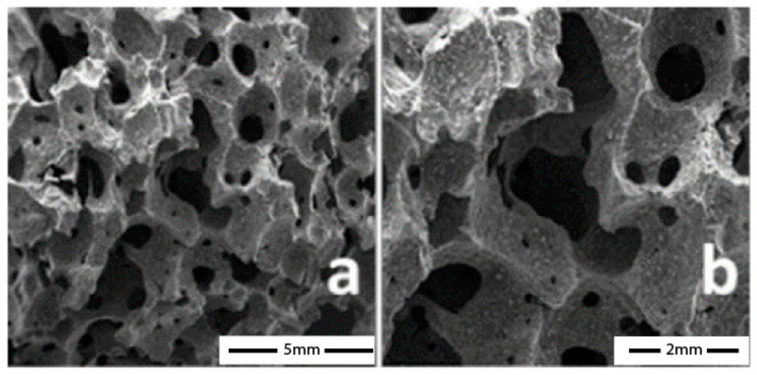
SEM micrographs report the microstructural characterisation of the porous geopolymer samples at 20× (**a**) and 40× magnification (**b**) [129].

SEM micrographs of the porous geopolymer are reported in Figure 18.

For in vivo testing on rabbits and biocompatibility analysis, the aluminosilicate material with the greatest porosity percentage and size range was chosen. In detail, geopolymers were implanted in femur bones (right femur as the positive control). Biopsies were carried out for histological analysis two and four weeks after implantation.

**Figure 19:**
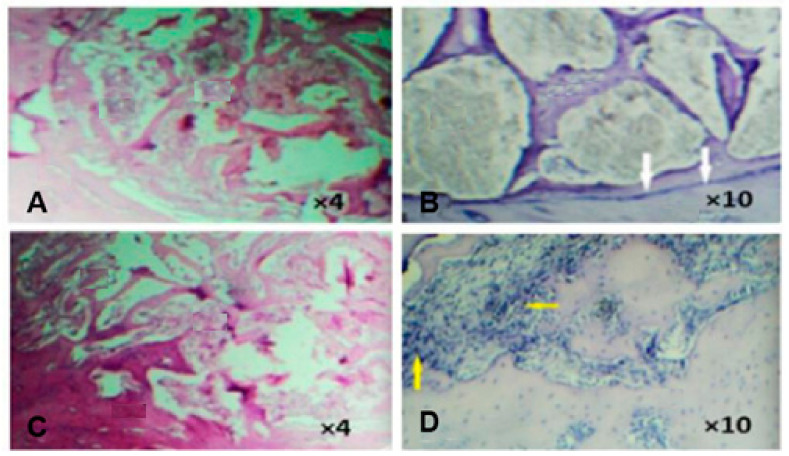
Optical images showing the presence of bone trabeculae units with the basal bone four weeks after surgery [(**A**) at 4× magnification and (**B**) at 10× magnification] and two weeks after surgery [(**C**) at 4× magnification and (**D**) at 10× magnification] [129].

Histological tests revealed that the implanted geopolymer material allowed the development of bone trabeculae with minimal inflammation. Figure 19 reports optical images of the samples that, after four weeks from the implantation, showed the presence of bone trabeculae units with the basal bone in several areas and had filled bone substitute material.

The authors concluded that foamed geopolymer systems improved bone formation compared to commercial bone substitutes. Thus, geopolymers could be considered promising scaffolds for bone substitutes, thanks to their availability and cost-effective characteristics.

**Figure 20:**
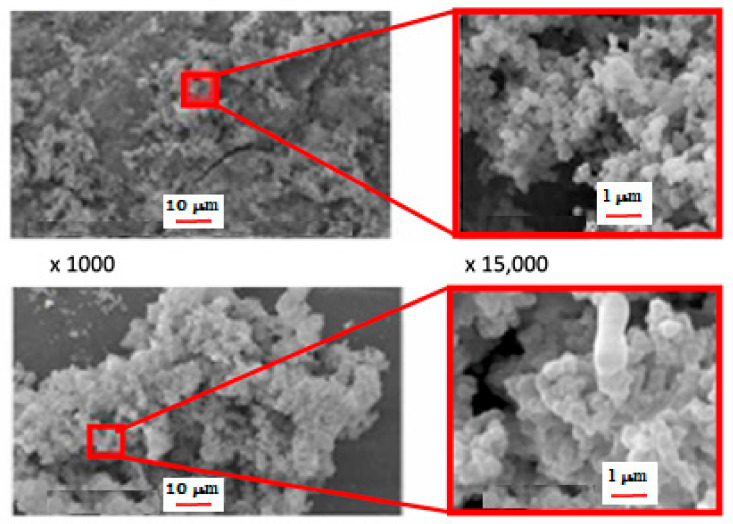
SEM micrographs at increasing magnifications of the same area reporting the microstructure of geopolymer material exposed to SBF solution for 28 days [130].

Mejíaet al. [130] prepared metakaolin- and CaCO_3_-based geopolymer materials and investigated their physical, mechanical, and biological (in vitro) properties. After seven days of curing, the ceramic materials displayed a percentage porosity between 50 and 30 and compressive strength between 18 and 29 MPa. The geopolymer sample with the highest compressive strength underwent a reactivity test for 28 days while exposed to simulated bodily fluid (SBF). SEM micrographs of the in-situ development of spherical crystals are presented in Figure 20. This enables the identification of components such as Ca and P.

**Figure 21:**
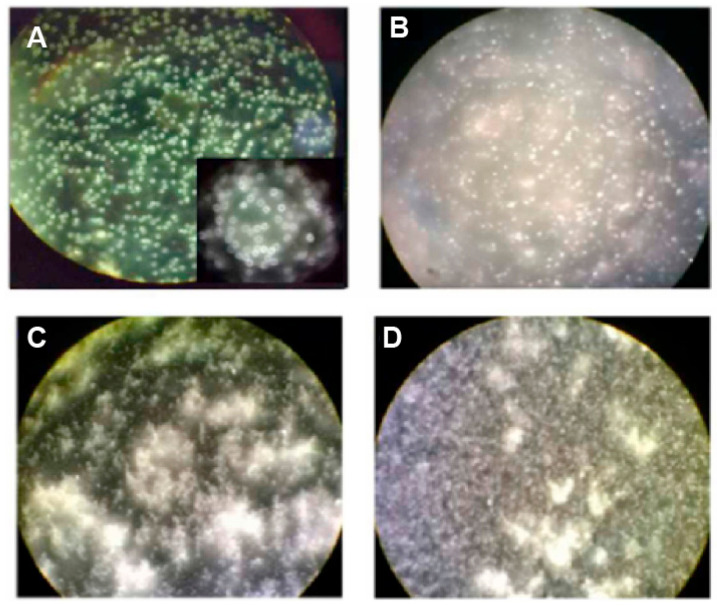
Microscopic images of haemolysis of the erythrocytes (globular morphological destruction and loss of oxygen transport ability) in blood samples in contact with different concentrations of ceramic discs: (**A**) (0 discs), (**B**) (1 disc), (**C**) (2 discs), (**D**) (3 discs) [130].

A geopolymer material that can develop a calcium phosphate layer on its surface was used for biological and antibacterial testing. In this instance, a 25 MPa compressive strength value and a rise in the pH of the SBF solution were found. Figure 21 displays microscopic pictures of the blood sample examination.

According to the findings of biological experiments, the high pH of the cellular environment damages red blood cells by causing them to burst. The authors concluded that the chemical composition of the specified geopolymer materials needs to be improved to reduce the pH value and the phenomenon of alkaline components seeping into bodily fluids. Finally, antibacterial tests have revalued the inhibitory activity towards Pseudomonas aeruginosa, indicating potential applications of geopolymer materials in external environments.

To investigate a Ti6Al4V alloy’s possible use as a composite material for prosthetic devices, Rondinella et al. [131] reported the creation of a thin and homogenous geopolymer covering it. Different geopolymer formulations (acidic and alkaline activation) were tested to maximise adhesion between the geopolymer coating and Ti6Al4V alloy, and multilayered coatings were added using the dip-coating technique. Scratch tests determined how well the geopolymer adhered to the metal substrate.

**Figure 22:**
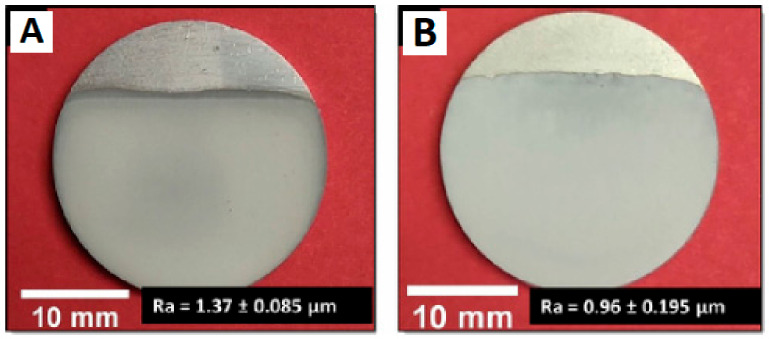
Optical images and roughness values of the geopolymer (obtained by alkaline activation) coating (**A**) and of the geopolymer (obtained by acidic activation) coating (**B**) [131].

The morphology and structuring of the geopolymer coating (obtained by alkaline and acidic activation reactions) are reported in Figure 22.

**Figure 23:**
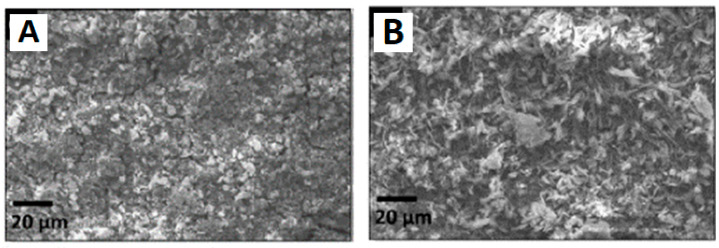
SEM micrographs of the geopolymer (obtained by alkaline activation) coating (**A**) and of the geopolymer (obtained by acidic activation) coating (**B**) [131].

The microstructures of the different kinds of geopolymer coatings (obtained by alkaline and acidic activation reactions) are reported in Figure 23.

After analysing its surface’s morphological and chemical features, a bacterial growth test confirmed the coating’s antibacterial properties.

According to the geopolymer coating’s microstructural, mechanical, and antibacterial characterisations, alkaline and acidic geopolymer coatings look structurally compact (although a few shrinkage cracks, especially in the acid-activated samples, can be seen in Figure 23) and seem to be suitable for biomedical applications.

Moreover, the fact that metakaolin was the most unreacted in the acid-activated geopolymer material indicates that these formulations have lower reactivity than alkaline ones. The acidic geopolymer coating was easily removed under light loads in scratch tests, while the alkaline formulation presented superior adhesion to the metal substrate. Both activation procedures’ coatings demonstrated comparable antibacterial properties, referring to the micro-organism’s growth.

### 4.3. Main Achieved Outcomes and Future Outlook for Geopolymer Materials

The analysis of literature data shows that significant research advancements in geopolymer materials such as bioscaffolds have been made in recent years [refs].

The main results achieved are as follows:

1. The starting aluminosilicate-based raw materials must be subject to careful characterisations to exclude the presence of toxic substances and harmful heavy metals.
2. A critical issue associated with geopolymer materials for biomedical applications is their high pH values, which could severely limit their biocompatibility. Geopolymers are generally obtained from the reaction of aluminosilicates with alkaline activating solutions (such as NaOH or sodium silicate). A helpful strategy to apply in this case is a high-temperature treatment of the hardened geopolymer matrix, which significantly reduces the geopolymer pH from 11.5 to 7.1 (physiological value).
3. The presence of “free” aluminium ions, which could cause significant toxicity, is a limiting factor for using geopolymers in bone tissue regeneration. A possible strategy is reducing the amount of aluminium involved in the reaction of geopolymerisation. This can be achieved with high-temperature treatments that involve the possible stabilisation of the free alkali present in the aluminosilicate matrix.
4. Geopolymers for biomedical applications should present a high porosity. It has been shown that highly porous geopolymers with a microstructure similar to bone tissue have increased biocompatibility while promoting more bone tissue growth.
5. A soaked-treatment method with a CaCl_2_ solution as an ion-exchange agent (after preparation, the samples were treated by soaking them in a CaCl_2_ solution for 24 h at 23 °C) enhances compressive strength and bioactivity by accelerating hydroxyapatite formation and slowing down the rise in pH.
6. The growth and nucleation of hydroxyl-apatite groups promote the biocompatibility of geopolymer materials. This phenomenon is improved when the materials contain cations, e.g., Na^+^, K^+^, or Ca^2+^ ions. Thanks to a cation-exchange mechanism, it is possible to observe the formation of hydroxyl-apatite [Ca_10_(PO_4)6_(OH)_2_], which is very important for improving the osteoconductive properties of geopolymer materials.

Future studies may aim to optimise formulations to achieve trabecular geopolymer structures with controlled porosity and high strength using an additive manufacturing process. This technique allows for obtaining several complex geometries and the high reproducibility of experimental conditions.

Durability studies of geopolymer scaffolds should also be conducted to understand the potential average lifetime of the geopolymer implant in the bone.

In addition, to improve the biocompatibility and osteoconductive properties of the geopolymer matrix, the research could be directed towards developing organic geopolymer-based composite materials with biopolymers.

The combination of a geopolymer matrix, which allows the obtaining of highly porous and mechanically stable systems, with biopolymers, which significantly increase biocompatibility and osteoconduction processes, could create the ideal material for bone tissue engineering.

## 5. Conclusions

Modern fracture treatment procedures have advanced to a high level of technology, ensuring the highest level of care. However, several clinical issues still have not been resolved, including flaws, bone loss, a lack of vascularisation, soft tissue injury, insufficient mechanical stability, infections, and malignancies. These issues are still major obstacles to effective bone repair.

The urgent need to develop innovative biomaterials to improve the healing processes of bone tissue using a reproducible, affordable, and low-environmental-impact alternative synthetic strategy has focused researchers’ attention on geopolymer materials thanks to their mechanical properties and structures with the capability to bond the bone matrix.

Although geopolymers exhibit ideal chemical–physical, mechanical, and morphological characteristics for use in BTE, their application in biomedical engineering still needs to be improved. The limitation in using these materials for bone tissue fabrication is mainly due to low biocompatibility and limited osteoconductive activity.

However, the growing interest in geopolymers as biomaterials in the BTE field in recent years allows for speculation that these systems can be suitably modified to make a important contribution to the field of biomedical materials in the near future.

## Figures and Tables

**Table 1:** Leading biomaterials, their properties, chemical compositions, and applications.

	Advantages and Disadvantages	Composition	Application Fields
Metals	**Pros:** - high mechanical properties - high fatigue resistance - Ductility **Cons:** - poor biocompatibility - stiffness - high specific weight - corrosion	Stainless, steel, CoCrMo, titanium, Ti5A14V, nitinol, nickel, platinum, tantalum	Orthopedic, orthodontic, cardiovascular
Ceramics	**Pros:** - good biocompatibility - chemical inertness - ductilityhigh compressive strength - corrosion resistance **Cons:** - low impulsive tensile strength - high specific weight - brittleness - not easy to process	Alumina, zirconia, hydroxyapatite, beta, tri-calcium phosphate, pyrolytic carbon	Orthopedic, orthodontic, cardiovascular
Polymers	**Pros:** - toughness - low specific weight - processability **Cons:** - low mechanical strength - degradabulity over time - deformabulity over time	Polymethylmethacrilate (PMMA), ultra-high molecular weight polyethylene (UHMWPE), polylactic acid (PLA), poly tetrafluoroethylene (PTEE), nylon, polyethylene, polyurethane, celluloid, cellophane, polycaprolactone (PCI), polyglycolic acid (PGA), polylactic acid (PLA), poly-lactic-co-glycolic acid (PLGA), polytethers including polyethylene glycol (PEG), polyvinyl alcohol (PVA) and polyurethanes (PUs)	Orthopedic, orthodontic, cardiovascular, breast implants, scaffold for soft tissues

## Data Availability

Not applicable.
